# Predictive coding and multisensory integration: an attentional account of the multisensory mind

**DOI:** 10.3389/fnint.2015.00019

**Published:** 2015-03-26

**Authors:** Durk Talsma

**Affiliations:** Department of Experimental Psychology, Ghent UniversityGhent, Belgium

**Keywords:** multisensory integration, mental model, predictive coding, attention, top–down, bottom–up

## Abstract

Multisensory integration involves a host of different cognitive processes, occurring at different stages of sensory processing. Here I argue that, despite recent insights suggesting that multisensory interactions can occur at very early latencies, the actual integration of individual sensory traces into an internally consistent mental representation is dependent on both top–down and bottom–up processes. Moreover, I argue that this integration is not limited to just sensory inputs, but that internal cognitive processes also shape the resulting mental representation. Studies showing that memory recall is affected by the initial multisensory context in which the stimuli were presented will be discussed, as well as several studies showing that mental imagery can affect multisensory illusions. This empirical evidence will be discussed from a predictive coding perspective, in which a central top–down attentional process is proposed to play a central role in coordinating the integration of all these inputs into a coherent mental representation.

## An Attentional Account of the Multisensory Mind

Imagine watching a rerun of the famous TV-series the Muppet Show. One popular character, the Swedish chef, is known for its gibberish fake Swedish, which at first appears not to make sense at all, other than its comical effect. Yet by carefully watching the Muppet’s mouth movements and the various additional cues given by bodily motions, it becomes suddenly clear that the fake Swedish is actually garbled English. As you watch the end of the clip, you can clearly understand the phrase “The chicken is in the basket” as the chef throws poor Camilla the hen through a basketball ring. Imagine now continuing with a different episode, and you may instantly recognize the chef’s response “The dog is in the pot,” in response to Miss Piggy’s query “what happened to my dog Foo Foo.”

This example clearly illustrates a major problem that we humans regularly have to overcome in interpreting sensory information, namely resolving ambiguities. Our understandability of the chef’s garbled fake Swedish is greatly enhanced through several non-verbal cues. These cues involve direct visual cues, such as the mouth movements accompanying his speech and several other visual cues that provide the appropriate context for understanding the scene. In addition, memory cues that are based on previous experience with similar scenes may also help us in our interpretation. But exactly how do we manage to integrate all these cues?

To answer this, the discipline of *Multisensory Processing* investigates the mechanisms contributing to the combining of information from our various senses. According to Stein et al. (2010), *Multisensory Integration*, refers to the neural process by which unisensory signals are combined to form a new product or representation. While multisensory processing studies have greatly increased our understanding of the processes directly involved in combining information from multiple senses, it is still not quite clear how our interpretation of sensory information can be enhanced by other sources of information, such as our existing background knowledge based on prior experience. In this review, I aim to discuss how these cues might be integrated with ongoing sensory input to generate a consistent mental representation.

## Multisensory Integration: Top–Down and Bottom–Up Processing

Our understanding of brain function has increased sharply in the last 20 years or so. In multisensory processing research we have equally witnessed a rather dramatic shift in our understanding of the processes that combine information across the individual senses. Before the seminal single cell recording studies in animals that demonstrated the existence of multisensory neurons in the superior colliculus (Wallace et al., 1998; Wallace and Stein, 2001), a predominant view in the late 1980s and early 1990s was that multisensory integration takes place relatively late in the processing stream, in cortical areas known as secondary association areas. For instance, in their influential late 1980s textbook “Brain, Mind, and Behavior,” neuroscientists Floyd E. Bloom and Arlyne Lazeron, write:

*“Association areas in the parietal lobe, for example, are thought to synthesize information from the somatosensory cortex–messages from the skin, muscles, tendons, and joints about the body’s position and movement–with information about sight and sound transmitted from the visual and auditory cortices in the occipital and temporal lobes. This integrated information helps us to form an accurate sense of our physical selves as we move through our environment.” (Bloom and Lazeron, 1988, pp. 274–275)*.

**FIGURE 1:**
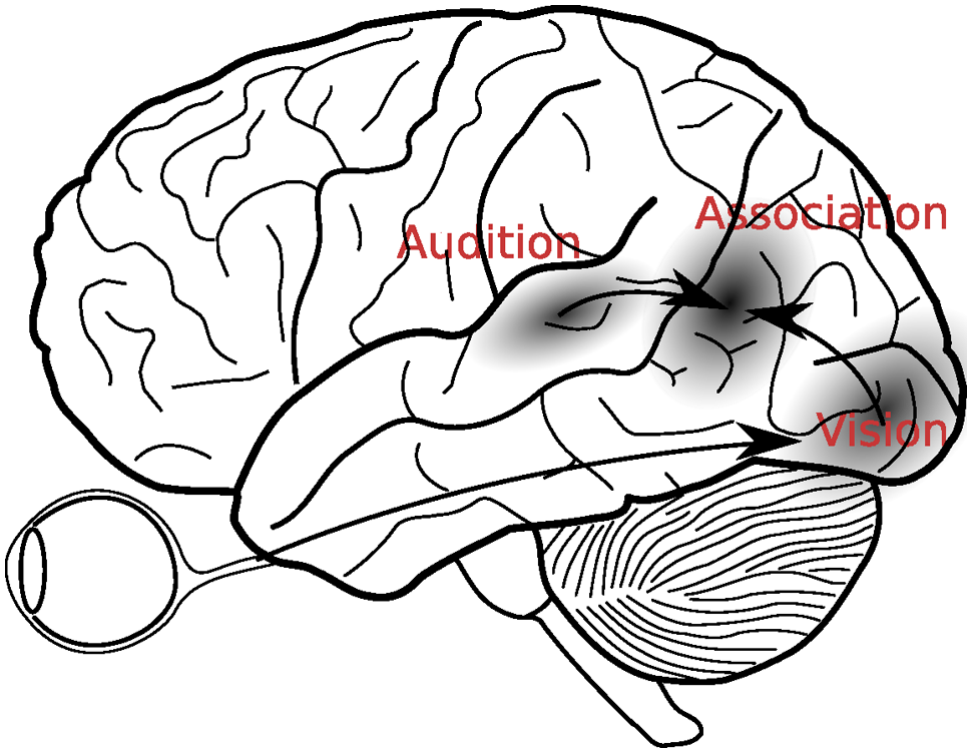
**A classical view of multisensory integration. According to this view, visual and auditory signals were first analyzed in the respective sensory cortices, before they were integrated in the secondary association areas, located in the temporo-parietal areas between the auditory and visual cortices**.

Bloom and Lazeron’s (1988) description clearly indicates that the merging of information across the senses was supposed to take place *after* the initial sensory processing had come to completion (see **Figure 1**). Since that time, however, many discoveries have suggested that multisensory integration is more complex than this. For example, in addition to the aforementioned single cell recordings, electrophysiological studies showed that multisensory interactions can already take place as early as 40 ms after stimulus presentation, which is considerably earlier than initially thought possible (Giard and Péronnet, 1999; Molholm et al., 2002).

### A Predictive Coding Account

One influential framework to explain the intricacies of multisensory processing is that of predictive coding. The predictive coding framework states that the brain produces a Bayesian estimate of the environment (Friston, 2010). According to this view, stochastic models of the environment exist somewhere in the brain^1^, which are updated on the basis of processed sensory information. These stochastic models (see Klemen and Chambers, 2011 for a review) thus provide the brain areas lower in the sensory processing hierarchy with predictions (or in Bayesian terms “priors”) that can be used to adjust the processing of ongoing sensory input. A strong mismatch between the prediction and the actual sensory input will then result in a major update of the internal model. For example, when unexpected sensory input is present, our internal model may require updating to deal with this change in representations.

### Predictive Coding: Top–Down vs. Bottom–Up Processing

The aforementioned mismatch is a typical example of bottom–up processing, in which sensory input adjusts the internal model. Conversely, our internal model may also affect the processing of our sensory information. This type of processing is known as top–down and is closely related to selective attention. For example, when we are in a complex environment with many stimuli competing for processing capacity, the most relevant ones need to be prioritized. This is possibly accomplished because the higher-order brain areas that are part of a fronto-parietal network can selectively bias the processing in the lower-order perceptual ones (Corbetta and Shulman, 2002). In other words, attending to task-relevant stimuli might be necessary for them to gain a stronger representation in our neural system.

Viewed within context of the predictive coding framework, the internal representation of our external environment is constantly updated on the basis of sensory input (i.e., forward connections). Sensory processing is, in turn, modulated on the basis of predictions provided by the active representations (i.e., backward connections). It can thus be argued that backward connections from the higher-order to the lower-order brain areas might embody the causal structure of the external world while forward connections only provide feedback about prediction errors to higher areas. In other words, anatomical forward connections are functional feedback connections, and vice versa (Friston, 2005). Mismatches, or –more formally, *prediction errors–* will thus result in strong adjustments in the internal representation and in strong top–down functional feedforward (or anatomical feedback) signals. One possible consequence of such a major prediction error is that the focus of attention shifts to a different aspect of the environment. Seen this way, attention could be considered as a form of predictive coding; a process that establishes an expectation of the moments in time when the relevant, to be integrated stimulus inputs are to arrive (Klemen and Chambers, 2011). It should be noted, however, that while attention may boost the precision of the predicted sensory input (and thus contributes to determining which aspects of our mental representation needs to be updated) the manifestations of attention and expectation can be radically opposite. Whereas expectancy reduces the sensory neural responses, attention enhances them, presumably due to heightening the weighting of the prediction error (Kok et al., 2011, 2012).

The closely related model of optimal Bayesian integration (Anastasio et al., 2000; Deneve and Pouget, 2004), has already been applied to a host of problems related to visual attention, in which attention is considered to provide the lower level visual cortices priors that serve to reduce stimulus ambiguity and therefore enhance the visual search process (Chikkerur et al., 2010). Additionally, it has been successfully applied to explain a number of basic multisensory processing phenomena (Ernst and Banks, 2002; Alais and Burr, 2004; Helbig and Ernst, 2007; Holmes, 2009). Despite these applications, the success with which Bayesian interference models can describe the interaction between attention and multisensory integration remains yet to be answered.

### Bottom–Up Principles of Multisensory Integration

Ongoing research that has investigated parts of this interaction may give us at least a partial answer, however. The information contained in the flow of input from the individual senses to the higher-order brain areas can, at least under certain circumstances, determine whether the stimuli contained in these streams are integrated or not. A large number of principles have been uncovered that explain under which conditions inputs to different modalities interact or not (Stein and Meredith, 1993; Noesselt et al., 2007; Stein and Stanford, 2008; Stein and Rowland, 2011). These principles were originally strongly related to the stimulus input characteristics, as well as the individual stimulus processing capabilities of each sensory modality (Stein and Meredith, 1993). For instance, input into the visual modality may influence spatial processing in the auditory modality, while input to the auditory modality may affect temporal processing in the visual modality; two characteristics that have been detailed in the modality appropriate hypothesis (Welch and Warren, 1980; Vatakis and Spence, 2007). Moreover, spatial and temporal proximity (Lewald and Guski, 2003), or the relative intensity (known as the “law of inverse effectiveness”; see Holmes, 2009) of multisensory inputs may be critical factors in determining whether two inputs integrate.

The near-simultaneous stimulation of two or more senses has also been shown to result in increased fMRI BOLD responses (Calvert et al., 2000; Fairhall and Macaluso, 2009) in several brain areas, including the superio-temporal sulcus, superior colliculus, and primary visual cortices. Moreover, increased early latency (∼40 ms after stimulus) event-related potential (ERP) responses to these stimuli (Giard and Péronnet, 1999; Molholm et al., 2002), better performance on stimulus identification tasks (Stein et al., 1996), and visual search benefits (Van der Burg et al., 2008, 2011; Staufenbiel et al., 2011; van den Brink et al., 2014) have been observed. Butler et al. (2012), used EEG recordings and a frequency mismatch paradigm to show that auditory and somatosensory cues elicit a multisensory mismatch, which indeed suggests that these cues can be combined pre-attentatively. Similarly Yau et al. (2009) showed that vibrating somatosensory stimuli for could affect frequency discrimination of auditory stimuli and vice versa (see also; Yau et al., 2010; Butler et al., 2011). Interestingly, however, amplitude judgments were not affected in this fashion. These results show that several stimulus features can automatically influence the processing of stimuli presented in another modality.

### Bottom–Up Integration Can Drive Attention

Several behavioral and ERP studies have shown that an object that is simultaneously detected by several sensory systems has a greater potential for capturing one’s attention (Van der Burg et al., 2008, 2011; Ngo and Spence, 2010; van den Brink et al., 2014). For instance, Van der Burg et al. (2008), showed that equally task irrelevant auditory stimuli could have strong beneficial impact on participants’ ability to detect visual target stimuli. In this study, a visual search task was used to show that a visual target stimulus that was not very salient by itself could become instantly noticeable when it was accompanied by a short tone. This result, labeled the “Pip and Pop” effect, suggests that multisensory stimuli are indeed able to capture attention and therefore that multisensory integration processes themselves operate pre-attentively. In a subsequent ERP study (Van der Burg et al., 2011) we showed that an early latency ERP effect, occurring around 40 ms over posterior scalp areas, correlated significantly with sound-induced performance benefits in this task. Moreover, we also found evidence that the sound integrated with the visual stimulus in a strongly automatic fashion: whenever a sound was presented, we observed an N2pc component in the ERP waveform, regardless of whether the corresponding visual stimulus was task-relevant or not. Since the N2pc is generally considered to be a neural correlate of automatic bottom–up attentional deployment (Luck, 1994), this can be taken as evidence for automatic integration of the sound with a corresponding visual stimulus. This further suggests that when a sensory modality is processing a stimulus simultaneously with one presented to another modality, these concurrently presented stimuli have a natural tendency to be processed in greater depth than stimuli that are either non-concurrent in time. Thus, these results not only suggest that bottom–up processes have a major influence on multisensory processing, they also show the involvement of early latency unisensory processes in multisensory integration.

### Top–Down Influences on Multisensory Processing

Interestingly, multisensory integration is not strictly guided by these principles. For instance Wallace et al. (2004) and Körding et al. (2007) have shown that even when low-level features of multisensory stimuli are perfectly matched, behavioral performance is impaired when these features are perceived to originate from two separate sources. Moreover, using vision and touch, Ernst (2007) trained participants to integrate arbitrary combinations of inputs and found that after training discrimination thresholds had increased for incongruent haptic/visual stimulus combinations. Likewise, Fiebelkorn et al. (2011) modulated the probability of co-occurrence of visual and auditory inputs. They found that hit rates for near-threshold visual inputs depended not only on the mere presence of the auditory input, but also on the participants’ expectation: hit-rates for simultaneously presented visual stimuli increased specifically when participants expected the auditory and visual inputs to be simultaneous. Additionally, it has been shown that a stimulus presented in one modality can affect the processing of an accessory stimulus presented in another modality, either due to its task relevance (Busse et al., 2005; Donohue et al., 2014), or because of learned associations between the individual inputs (Molholm et al., 2007; Fiebelkorn et al., 2010). For instance, Busse et al. (2005) paired task-irrelevant auditory stimuli with either attended or unattended visual stimuli and found that processing of the tones that were paired with attended visual stimuli started to differ from that of the tones paired with unattended visual stimuli, around 200 ms after stimulus onset, as expressed by a difference in the ERP waveforms, suggesting that attentional processes in the visual modality strongly affected the processing of irrelevant stimuli in the auditory modality. This difference, which subsequently has been interpreted as a multisensory late processing negativity (Talsma et al., 2007), was found to originate in the primary auditory cortex, as shown using fMRI (Busse et al., 2005; Experiment 2). Thus, these results show that top–down factors can strongly influence multisensory processing.

Following the notion that top–down processing influences multisensory perception, a profound number of recent studies has shifted focus from uncovering the aforementioned basic principles of multisensory integration, to investigating how these principles interact with other cognitive processes. For example, the principle that stimuli are more likely to be integrated when they overlap in space has been found to be more task-dependent than originally assumed (Cappe et al., 2012) and the necessity for temporal correspondence has also been found to depend on tasks and stimulus type (van Atteveldt et al., 2007; Stevenson and Wallace, 2013). These recent findings are somewhat reminiscent of earlier work by Lewald and Guski (2003) who have shown that ones’ belief that two stimuli have a common cause might affect whether we perceive cross modal inputs as being one integrated stimulus, or as multiple ones (see also Welch, 1999 for a similar suggestion). Additionally, processes such as attention (Tiippana et al., 2004; Alsius et al., 2005, 2007, 2014; Senkowski et al., 2005, 2007; Talsma and Woldorff, 2005; Talsma et al., 2007; Mozolic et al., 2008; Hugenschmidt et al., 2009) or memory (Thelen et al., 2012) have been shown to affect multisensory processing.

### The Multifaceted Interplay between Attention and Multisensory Processing

Thus, it appears that the automaticity of multisensory integration depends on a variety of factors: If the individual stimuli in this bottom–up stream are in themselves salient enough, they can be integrated; specifically when they are approximately matched in time and location with a stimulus processed in another modality. If they are not salient enough, additional prioritizing by an attentional mechanism may be needed (Talsma et al., 2010), suggesting that multisensory integration involves both top–down and bottom–up processes.

If multisensory integration is the result of a complex interaction between top–down and bottom–up processes, then it should take place at multiple stages of processing. So, are we able to identify these stages? Several human electrophysiology studies have shown that *multisensory interactions* can occur at latencies that would exclude the possibility that multisensory processing only occurs after initial sensory analysis has come to completion (Giard and Péronnet, 1999; Molholm et al., 2002; Van der Burg et al., 2011). These interactions indicate that information from one sensory modality can influence the information processing in another one, without necessarily forming a new mental representation. The aforementioned studies thus indicated that although the primary and secondary (uni-) sensory brain areas are possibly involved in multisensory processing, these multisensory processes do not necessarily result in a newly integrated representation. These studies do suggests, however, that multisensory processing is intertwined with basic sensory analysis in a much more intimate fashion than previously thought possible.

To summarize, the findings discussed above show that multisensory integration depends to a much smaller degree on rigid bottom–up principles than originally believed to be the case. By contrast, they show that multisensory integration is by a very large factor determined by top–down processes. The next question now is, how these top–down and bottom–up processes interact, and at which processing stages this occurs.

## Early and Late Accounts of Multisensory Processing

As has become clear by now, since the beginning of the 1980s, our understanding of multisensory processing has shifted from relatively rigid and principle-based mechanisms, located late in the processing stream, to a highly flexible process consisting of multiple stages. At least a two sub-processes, one of which can occur very early on in the processing stream, have been identified (Calvert and Thesen, 2004; Talsma et al., 2007; Koelewijn et al., 2010; Baart et al., 2014). In spite of this change in interpretation, there are still a number of arguments to not completely discard the original idea that multisensory integration (partially) takes place after the initial sensory processing has come to completion. It has recently been proposed that the integration of neural representations is an intrinsic property of the brain (Ghazanfar and Schroeder, 2006; van Atteveldt et al., 2014). From this idea it follows that different levels of neural interactions may take place at progressive levels of processing of the sensory inputs.

### Multisensory Integration: An Intrinsic Property of the Brain?

van Atteveldt et al. (2014), have suggested that multisensory integration is a process that operates on the basis of the flexible recruitment of several general purpose brain functions that are thought to synchronize activation within several neural pathways. These pathways are thought to connect the sensory cortices, either directly to each other (Falchier et al., 2002), or through cortico-thalamic-cortical pathways (Hackett et al., 2007; Lakatos et al., 2007; van den Brink et al., 2014), suggesting that information can be transferred relatively directly between these brain areas. Another set of these pathways involves recurrent feedback projections from the frontal cortex (notably the frontal eye fields and ventral prefrontal cortex). It is assumed that these feedback mechanisms coordinate activation in the sensory cortices through attention. The general idea is that these recurrent feedback projections can send biasing signals to the perceptual brain areas. The feedback signals can then induce an increase in sensitivity in neurons responsive to the attended feature, while simultaneously causing a decrease in sensitivity of neurons not responsive to the attended feature (Motter, 1994; LaBerge, 1995; Corbetta and Shulman, 2002). This attentional bias can either be expressed overtly, that is, by actively scanning the environment with the oculomotor system, or covertly by scanning the environment using selective attention only. Given the importance of these latter recurrent feedback connections, attentive scanning of the environment is an essential prerequisite for multisensory integration to take place.

A possible function of the direct and cortico-thalamic connections between visual and auditory cortex is that they enable cross-referencing between these cortices. In other words, auditory cortex receives advance information regarding visual processing and vice versa. Viewed from a predictive coding framework, prediction errors in the auditory representation are minimized by additional information presented by the visual system, and vice versa. In our own framework (Talsma et al., 2010), these low level interactions can for instance result in spatio-temporal realignment of the auditory and visual input signal. Thus, the early latency processes appear to cross-feed low-level information between the individual sensory cortices. This cross-feeding may modify the original input signal and can therefore be described as a multisensory interaction, but not necessarily as multisensory integration. Additional research is still required, however, to determine the exact functional role of these direct connections.

## Task and Stimulus Type Dependencies

### Task Relevance

To further differentiate between early and late multisensory processes, we need to distinguish between two rather strongly differing sets of research. Studies using relatively simple stimuli, such as beeps, and flashes, have predominantly focused on determining the bottom–up driven effects of multisensory processing that have been discussed in detail above (van Wassenhove et al., 2007; Stein and Stanford, 2008; Holmes, 2009; Noesselt et al., 2010; Rach et al., 2011). Studies using more naturalistic, meaningful stimuli, on the other hand, have more strongly emphasized the influence of top–down processing in multisensory integration. For instance, studies using speech fragments and movie clips have indicated that semantic congruence between visual and auditory stimuli also strongly influences multisensory processing (McGurk and MacDonald, 1976; Calvert et al., 2000; Tuomainen et al., 2005; Cappe et al., 2012). Most notably, the McGurk effect, that is, the illusion that speech sounds are being perceived differently when they are combined with non-matching lip-movements is one of the hallmarks of the effectiveness of multisensory integration. It has long been thought that this illusion is highly automatic, although that notion has been challenged by showing that one’s susceptibility to the McGurk illusion falters under high attentional demands (Alsius et al., 2005).

### Top–Down Effect in Multisensory Speech Perception

The involvement of semantic congruence in multisensory integration in speech processing presumably indicates that access to semantic information constrains the possible interpretation of the bottom–up auditory and visual input streams in a top–down fashion. In other words, access to prior knowledge may restrain the possible interpretations of both the visual and auditory input streams, which may in turn improve the segmentation of the auditory speech signal. Speech signal segmentation is generally problematic (specifically under noisy conditions; see Ross et al., 2007; Foxe et al., 2015), because there is only a very loose connection between speech sounds and the underlying phoneme structure (Liberman et al., 1967). Thus, constraining possible interpretations of the speech signal through top–down processes may further benefit from limitations imposed by information arriving from other modalities. Because of this, speech perception has been considered to be an intrinsic multisensory phenomenon (Stevenson et al., 2014) and it has even been argued that audio-visual speech perception is a special form of multisensory processing (Tuomainen et al., 2005; but see Vroomen and Stekelenburg, 2011).

### Speech Perception and Prior Experience

Interestingly, Vroomen and Baart (2009) showed that speech processing can be affected by lip-reading, but only when their participants could interpret the auditory stimuli as speech signals. This was done by dubbing sine-wave speech (Remez et al., 1981) onto video recordings of lip-moments. One interesting characteristic of sine-wave speech is that to most naïve listeners it sounds just like random sounds from a science fiction movie. Once participants get into speech mode, that is, once they start recognizing the sounds as speech, they usually never fail to ignore the speech component, much in the way that the realization that the Swedish Chef from our example speaks garbled English greatly enhances our comprehension of him. This point thus illustrates that prior experience and background knowledge may influence multisensory processing; a topic that will be discussed in more detail below.

### Multiple Stages of Multisensory Integration

Following up on this study, Baart et al. (2014), used ERPs to identify two distinct stages of multisensory integration in the processing of sine-wave speech. The auditory N1 component, a negative component about 100 ms after stimulus onset, peaked earlier for audiovisual stimuli than for auditory stimuli alone, regardless of whether participants were in speech mode or not. By contrast, the P2 component, a positive component peaking at roughly 200 ms after stimulus onset, was also modulated the presence of visual information, but only when participants were in speech mode. It should be noted that the latency of these latter ERP findings, while representative for speech stimuli (e.g., van Wassenhove et al., 2005), occurred somewhat later compared to those typically found in studies using simple beeps and flashes (Giard and Péronnet, 1999; Molholm et al., 2002; Senkowski et al., 2005, 2007; Talsma and Woldorff, 2005; Talsma et al., 2007; Van der Burg et al., 2011). It appears that the N1 component reflects a relatively automatic bottom–up process, while the P2 component reflects a process that is also affected by top–down processing. Despite this difference in latency, the notion of a two-stage approach in multisensory processing is compatible with earlier notions showing separate stages of multisensory processing for simple stimuli (Talsma et al., 2007).

Further evidence for the hypothesis that both top–down and bottom–up processing contribute to multisensory speech processing is provided by an fMRI study from Miller and D’Esposito (2005). These authors presented audiovisual speech fragments in which the relative onsets of the auditory speech stimulus were shifted with respect to the onset of the visual stimulus. Synchronous presentation of the auditory and visual speech signals resulted in a significantly larger activity in several brain areas that are involved in multisensory processing. These areas include Heschl’s gyrus, the superior temporal sulcus, the middle intraparietal sulcus, and the inferior frontal gyrus. The involvement of these brain areas provides more evidence that multisensory interactions occur at various stages of processing.

### Top Down Processing: Exclusively for Speech Stimuli?

**FIGURE 2:**
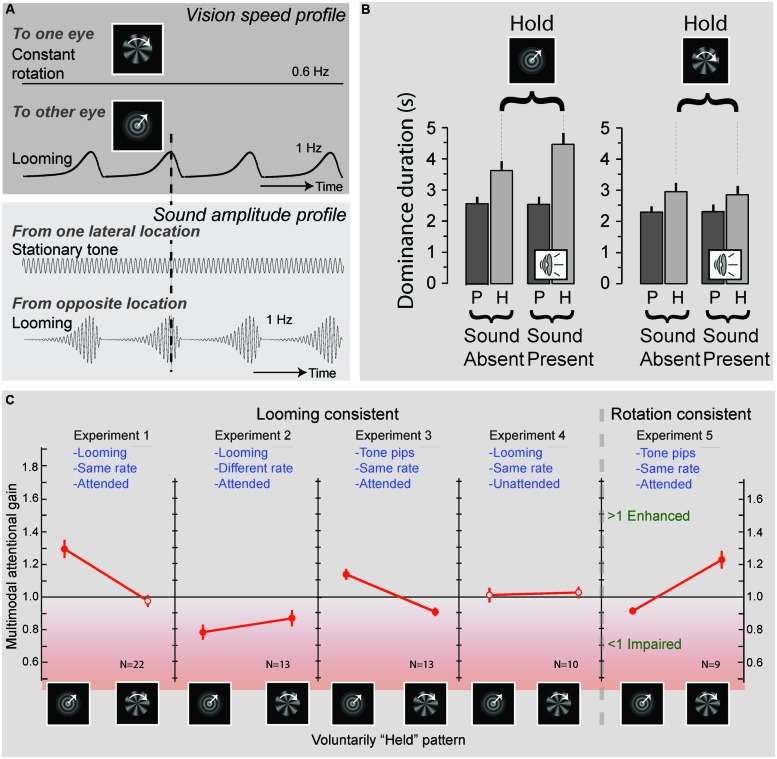
**Effects of attention and multisensory integration on conflict resolving in binocular rivalry**. **(A)** Experimental design: an object rotating at a frequency of 0.6 Hz was presented to one eye, while a looming object, expanding at a rate of 1 Hz, was presented to the other eye. Concurrent with the presentation of these visual objects sounds could be presented, consisting of, a stationary “e-chord” sound that was presented to one channel of a headphone, while a looming sound that matched the temporal characteristics of the looming object was presented to the other channel. Participants were required to attend to the looming sound pattern and report when the dominant visual pattern switched from the looming to the rotating image and vice-versa. **(B)** Average durations of the looming (left) and rotating (right) visual patterns being dominant. Duration times were significantly increased when participants were requested to attend and hold on to one of the patterns. Importantly, when the sound pattern was present this effect was enhanced for the (rhythmically congruent) looming visual pattern, but not for the (rhythmically incongruent) rotating visual pattern. These results suggest that attention can affect visual dominance by way of interacting with congruent sound patterns (P, passive viewing; H, hold on to instructed pattern). **(C)** Effects of rhythmic congruency and attention. Experiments 1–4 tested the influence of sounds that were consisted with the looming patterns. Experiments 1 and 3 show an increase in attentional gain (i.e., a prolonging in duration of the held pattern) when a sound was present that was rhythmically congruent with the held pattern. When the sound was rhythmically incongruent (Experiment 2) a decrease in attentional gain was observed, and when the sounds were unattended (Experiment 4) no significant change in attentional gain could be observed. Experiment 5 generalizes the results to rotating visual patterns. Filled red circles indicate attentional gains that significantly deviated from one. Adapted from van Ee et al. (2009) by permission of the Society for Neuroscience.

The processing of naturalistic audiovisual stimuli involves both top–down and bottom–up processing. This could lead one to conclude that whereas simple stimuli involve mostly bottom–up processes, complex (speech) stimuli involve both top–down and bottom–up processes. Upon closer inspection, however, this is probably overly simplistic. For example, by using a binocular rivalry paradigm that consisted of a visual stimulus containing looming motion presented to one eye, and radial motion to the other, van Ee et al. (2009) demonstrated that participants were able to hold on to one of the two percepts longer by means of attention. Interestingly, this attentional gain for one of the percepts was prolonged when the attended visual stimulus was accompanied by a sound that matched the temporal characteristics of the attended visual stimulus (**Figure 2**). This pattern of results also suggests a complex interaction between attention and multisensory integration. Although the exact neural mechanisms involved in this process are not yet fully understood, it appears that attention boosts the neural response to one of the competing visual signals, and that this boost, in turn, facilitates integration with the matching auditory signal. This finding suggests that rhythmic congruence between visual and auditory stimuli is another critical principle for multisensory processing. Interestingly, van Ee et al. (2009) also demonstrated that the mere presence of such a matched sound was insufficient. Instead, attention to both the visual and auditory modalities was needed to facilitate attentional facilitation of one of the two percepts. This result shows that multisensory interactions can influence visual awareness, but only in interaction with attention, underscoring that attention plays a pivotal role in multisensory processing.

## A Multisensory Representation

The evidence discussed so far shows that information from our various senses fuses at several stages of processing. Additionally, several studies show that multisensory speech processing can be affected by prior experience (Vroomen and Baart, 2009; Navarra et al., 2010; Vroomen and Stekelenburg, 2011; Nahorna et al., 2012). The next question is whether the influence of prior experience is limited to specific forms of speech processing or whether it can be generalized across multiple domains of multisensory processing.

### Prior Experience and Multisensory Memories

Thelen et al. (2012) have provided evidence for the role of memory in the formation of a multisensory representation. In this study, participants were required to memorize visual line drawings. These drawings were either presented simultaneously with a meaningless sound (multisensory context), or in isolation (unisensory context). One of the key findings of this study was that recognition accuracy was significantly impaired when the pictures had initially been presented in a multisensory context. This result suggests that the multisensory context provided in the initial presentation has become part of the mental representation. Thelen et al. (2015), subsequently manipulated the semantic relation between the visual and auditory stimuli, and also investigated the effects of multisensory memory formation on auditory processing. We found that auditory object discrimination was enhanced when initial presentations entailed semantically congruent multisensory pairs, and impaired when they entailed incongruent pairs, compared to sounds that had been encountered only in a unisensory manner. This result shows that the subsequent processing of a sensory trace is greatly affected by the initial context in which it was encoded. More specifically, a congruent pair of audiovisual stimuli may facilitate subsequent recall, whereas incongruent, or unrelated auditory and visual stimulus pairings may actually impair such recall. Thus, an internally consistent multisensory stimulus may be remembered more effectively than one that is internally inconsistent (see also Molholm et al., 2007; Fiebelkorn et al., 2010 for evidence of a similar role of learned multisensory associations in attention orienting and; Quak et al., in revision for a literature review on the relation between working memory and multsensory processing).

From a predictive coding perspective, semantically congruent audiovisual stimuli will result in higher-order brain areas receiving consistent information, which will result in a strong and consistent internal model, and a low prediction error. If the information presented is incongruent across modalities, this may result in an inconsistency in the internal model, which in turn may result in a greater error signal being sent back to the sensory cortex, which in turn may result in more effort being invested in encoding the representation, combined with a weaker internal representation.

Following the logic laid out by the predictive coding framework, one would also expect that stimulating only one sensory modality at the time will result in activation of another sensory modality. If, for example, we only present a picture of the Swedish Chef, our background knowledge may strongly affect the internal representation that we derive from this picture. Because his gibberish Swedish is such a characteristic feature, merely presenting an image of the muppet may not only activate our visual representation, it may also activate all concepts related to the Swedish Chef that we have gained through prior experience, including his characteristic manner of speaking. Depending on how strong these associations are, the image may not only trigger one’s knowledge of the Chef’s manner of speaking, it may even actively trigger recall of specific fragments, such as those given in the examples at the beginning of this article. Likewise, the presentation of a speech fragment may trigger similarly vivid mental images of the Chef’s characteristic manners of behavior. In terms of predictive coding, the internal representation would be activated because the sensory input signal matches with an existing representation stored in long-term memory. The resulting internal modal then not only projects feedback information to the original sensory cortex that activated the representation, but also to the other sensory cortices. If this assumption is correct, then we might expect that stimulating one sensory cortex, such as the visual one, might also result in activation of other sensory cortices, such as the auditory one. Next we turn to a number of studies that have provided evidence for this.

### Top–Down Induced Sensory Cortex Activation via Mental Imagery

Evidence for the idea that visual cortex might be activated indirectly by auditory stimuli comes from at least two different recent studies. Mercier et al. (2013) used intracranial recordings to show that auditory phase reset, and auditory evoked potentials can be recorded in the visual cortex. This study thus illustrates cross-sensory phase reset by a non-primary stimulus in “unisensory” cortex. Vetter et al. (2014) used fMRI to show that visual cortex was activated either by auditory stimuli, or by imagined stimuli. According to the Vetter et al. (2014). study, sound is initially processed through the classical auditory pathways. The resulting representation causes object-specific neural activation patterns that are subsequently projected back to visual cortex. Interestingly, sounds belonging to two different categories were correctly classified on the basis of the activation pattern observed in visual areas, regardless of whether this pattern was induced by a physical sound or by a mental imagery instruction, suggesting that higher-order cortical networks mediated the visual cortex activation. Vetter et al. (2014) further compared the sound-induced activation patterns with the imagery-induced activation in auditory cortex, but no similarities were found here. Moreover, they compared activation across different exemplars and different classes of stimuli and found that the information projected back appears to convey higher-level semantic information, as was shown using a multivariate pattern analysis. Finally, they found similar activation in multisensory convergence areas, including the precuneus and superior temporal sulcus, suggesting that the visual cortex activation was mainly induced by way of feedback from those multisensory areas.

These results are compatible with recent studies by Berger and Ehrsson (2013). These authors replicated two classical multisensory integration effects, but instead of presenting actual auditory stimuli, they instructed their participants to imagine these stimuli. The cross-bounce illusion consists of two circles moving on a collision course (Sekuler et al., 1997). Phenomenally, the two circles can either be perceived as crossing each other, after making contact, or as bouncing off of each other. When a sound is presented at the moment of contact, participants tend to report more often that two stimuli bounce instead of continuing on their original course. Interestingly, Berger and Ehrsson (2013) showed that these effects could occur not only using actual auditory stimuli but also using imagined ones, a result that is consistent with the Vetter et al. (2014) conclusion that auditory imagery can affect visual processing. Similar results were also found for an imagined version of the McGurk illusion.

In a second experiment, Berger and Ehrsson (2013) also showed that visual imagery can affect auditory processing. This was done using an imagery version of the ventriloquist effect. The ventriloquist effect describes how a sound can be mislocalized because it coincides with a visual stimulus that is presented at a different location. In the imagery version of the ventriloquist effect, participants imagined the presence of a circle at a specific location. Participants’ estimates of the location of sounds presented at nearby locations were systematically biased toward the location of the imagined stimulus. In a subsequent fMRI study (Berger and Ehrsson, 2014), it was found that simultaneous visual imagery and auditory stimulation resulted in an illusory translocation of auditory stimuli that was associated with activity in the left superior temporal sulcus, a key site for the integration of real audiovisual stimuli (Driver and Noesselt, 2008; Ghazanfar et al., 2008; Dahl et al., 2009; Beauchamp et al., 2010; Nath and Beauchamp, 2012). These findings show that processing in brain areas that we considered until recently to be unisensory can be influenced by a variety of sources. This malleability of the sensory cortices can possibly also explain why enhanced peripheral visual processing can be observed in congenitally deaf participants (Scott et al., 2014). Scott et al. (2014) used fMRI to find that congenitally deaf patients show better peripheral vision, a change that is presumably supported by a reorganized auditory cortex. More specifically they found that this increase in peripheral vision related to changes in sensitivity in Herchl’s Gyrus, as well as several other visual and multisensory areas, including the posterior parietal cortex, frontal eye fields, anterior cingulated cortex, and the supplementary eye fields. In addition to the already established direct links between the sensory cortices (Falchier et al., 2002; van den Brink et al., 2014), the results discussed in the previous section show that one sensory cortex can also activate another one via a slower route involving higher cortical areas that provide feedback at a more abstract level.

## An Attentional Account of the Multisensory Mind

We have seen that the cortical areas that until recently were characterized as “unisensory” are far more interconnected than previously thought possible. The final question is how the interplay between all these connections can result in multisensory integration.

**FIGURE 3:**
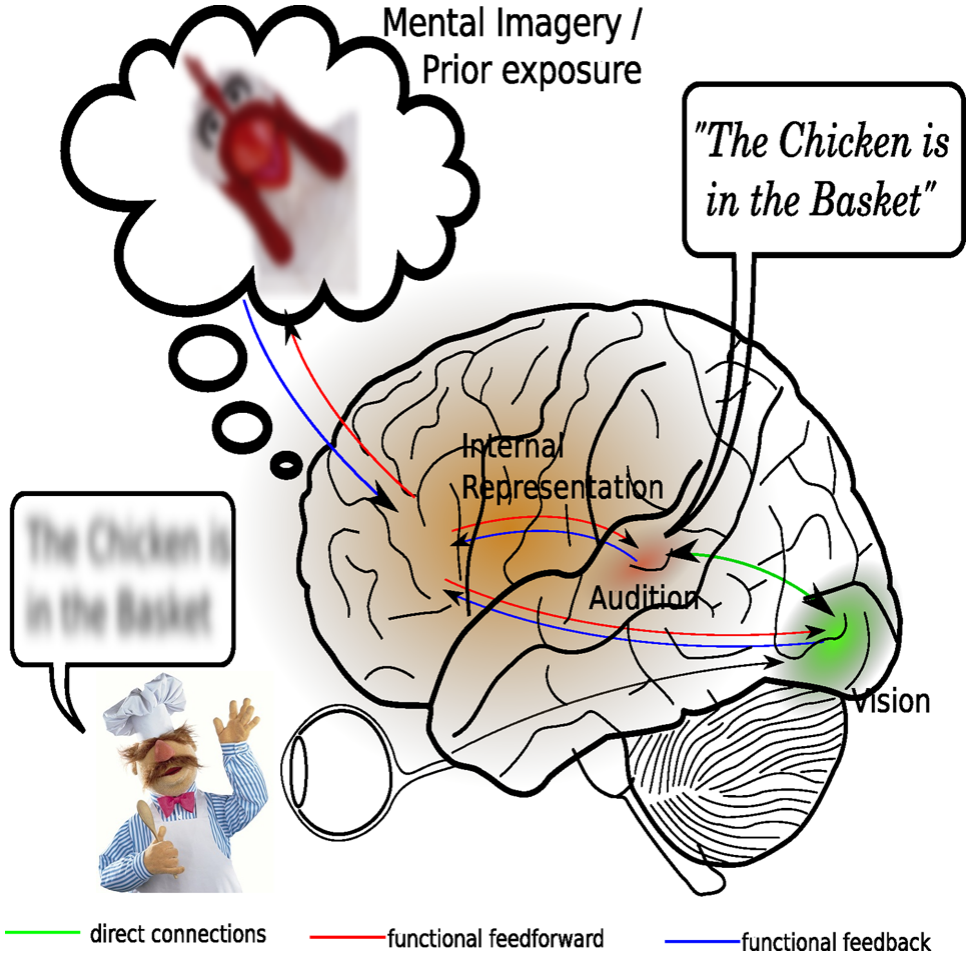
**An attentional account of multisensory integration. Central in this revised view of multisensory integration is the presence of a dynamic mental representation, which is updated on the basis of sensory inputs as well as on the basis of representations stored in memory**. Shown here is an example of how inaudible speech may benefit from both direct visual stimulation, as well as from the context provided by prior exposure to a similar situation. Processing in the visual and auditory sensory cortices is depending on expectancies generated by the internal models. A mismatch between expected input and actual input, formally known as a prediction error, may result in enhanced activation in the sensory cortices. Multisensory integration here is considered to consist of synchronization of activity in the auditory and visual cortices. This integration is facilitated by direct and thalamo-cortical connections between the auditory and visual cortical areas.

According to the predictive coding framework, mental representations of our external environment are actively constructed by our higher-order brain processes (Friston, 2010), on the basis of sensory input and our existing background knowledge (**Figure 3**). Moreover, these mental representations serve to form predictions about future changes in the external environment so that sensory processing is optimized to predominantly deal with unexpected changes (Baess et al., 2011). Given that backward connections might embody the causal structure of the external world while forward connections only provide feedback about prediction errors to higher areas, it can be argued that both types of connections are needed for integration. The higher-order brain areas containing the conceptual representation provide functional feedforward information to the sensory cortices. Viewed this way, multisensory integration actually takes place because an attentional mechanism combines the information contained in the existing mental representation with general background knowledge and uses the resulting model to update sensory processing, much in the way that attention has been proposed to bind together several stimulus feature within the visual modality (Treisman and Gelade, 1980). Viewed this way, it can be tentatively stated that multisensory integration is largely accounted for by attentional mechanisms.

Although this view actually places multisensory integration again at the end of the processing stream from an anatomical perspective, it does not exclude the possibility of multisensory interactions occurring early on in the processing stream. A mismatch between the senses can, depending on the complexity of the input stream, be resolved in the relatively early stages of processing, presumably involving the aforementioned direct or cortico-thalamo-cortical pathways. Presumably, the processes involved here are mainly bottom–up. Although these early sensory interactions may take place at early stages, it should be noted that, following the logic of the predictive coding framework, the individual sensory representations serve nothing more than to update the internal model represented in the higher-order brain areas. It is plausible that the individual senses interact before they update our mental representation, because reducing ambiguities in the individual input channels will inevitably result in a reduction of prediction errors that need to be fed-back to the sensory cortices. Although multisensory interactions can thus take place early on in the processing stream, their presence does not necessarily indicate multisensory integration.

## Summary and Conclusion

Despite the initial idea that sensory information integrates in higher-order association areas of the neocortex, substantial amounts of evidence now point toward a much more diffuse process, in which multisensory operations can take place at various stages of processing. Moreover, multisensory processes can be affected by a host of other cognitive processes, including attention, memory, and prior experience.

More generally, this literature review has shown that multisensory processing and attention are strongly related to each other. This brings us to the question whether the role of attention in multisensory integration is a matter of bottom–up or top–down processing. Though speculative, I would argue that while multisensory processing in general involves both bottom–up and top–down processes, the more specific case of multisensory integration is largely subserved by top–down processes: From a predictive coding point of view, it can be argued that integration takes place because higher-order networks actively maintain a mental model of the environment, which generates predictions about the expected sensory input. Sensory processing itself is adjusted on the basis of the (dis)agreement, between the actual sensory activity and the activity predicted by the model. Moreover, this prediction error may also require an update of the model itself. According to this view, it is in this mental model where sight, sound, smell, taste, and touch is integrated with our existing cognitive schemata. Several lines of evidence support this idea; early bottom–up driven processing in one modality can subsequently modify the internal representation of a stimulus in another sensory modality (Busse et al., 2005; Van der Burg et al., 2008, 2011), suggesting that functional feedback from the sensory system results in a change in prediction of another sensory modality. Additional influences from prior experience (Vroomen and Baart, 2009; Thelen et al., 2012, 2015, or mental imagery also actively affect multisensory processing (Berger and Ehrsson, 2013, 2014). Moreover, evidence exists to show that such imagery can, just like actual sensory input, activate processes in another modality (Vetter et al., 2014). Because the processes that are involved in integrating the inputs from such a wide variety of sources are essentially top–down and bearing a strong resemblance to attentional control mechanisms (van Atteveldt et al., 2014), it can be argued that attention plays an essential role in integrating information. Seen this way, attention counts as an essential cognitive faculty in integrating information in the multisensory mind.

## Footnotes

1 Given that the predictive coding framework essentially describes a hierarchy of processing levels, it does not identify specifically which brain areas are involved in creating these internal models. In general, the higher the level of processing, the more distributed the network of brain areas involved can become. For instance, Rao and Ballard (1999) presented a predictive coding model of receptive field effects in striate cortex and stated that, in their simulations, the “internal model is encoded in a distributed manner within the synapses of model neurons at each level.” Although it is not yet understood how stochastic models of the environment are coded in the brain, seminal work by Tolman (1948) has shown that cognitive maps can be formed on the basis of relations among salient cues and that these representations support navigation and inferential expression. Subsequently, these maps have been linked to hippocampal functions (see e.g., Morris et al., 1982). The hippocampal and prefrontal cortex interact with each other in decision making (Yu and Frank, 2015). The prefrontal cortex is thought to contain a hierarchical structure of mental representations (Badre, 2008), which in turn are connected to the parietal cortex. This network bears some resemblance to the network of brain areas involved in selective attention (Corbetta and Shulman, 2002) and could jointly support the formation of higher-order stochastic models. This conjecture is speculative, however, and further research in this area is needed.
